# Adequacy of nutrients important for brain health and dietary supplement use among older adults in the U.S. POINTER trial

**DOI:** 10.1016/j.jnha.2026.101026

**Published:** 2026-11-04

**Authors:** Christy C. Tangney, Marjorie Howard, Jennifer Ventrelle, Puja Agarwal, Katelyn R Garcia, Sarah M Graef, Thomas Holland, Masun Jackson, Jeffrey Katula, Joseph Masdeu, Talitha Baszlie, Kristie Miller, Rose A. Trevino-Whitaker, Heather M. Snyder, Laura D. Baker, Mark A. Espeland, for the U.S. POINTER study

**Affiliations:** a Departments of Clinical Nutrition, Rush University Medical Center, Chicago, IL, United States of America; b Department of Family & Preventive Medicine, Rush University Medical Center, Chicago, IL, United States of America; c Department of Internal Medicine, Wake Forest University School of Medicine, Winston Salem, NC, United States of America; d Department of Internal Medicine, Rush University Medical Center, Chicago, IL, United States of America; e Department of Undergraduate Studies, Rush University Medical Center, Chicago, IL, United States of America; f Advocate Aurora Research Institute, Park Ridge, IL, United States of America; g Department of Health & Exercise, Wake Forest School of Medicine, Winston Salem, NC, United States of America; h Department of Neurology, Houston Methodist Hospital, Houston, TX, United States of America; i Kelsey Research Foundation, Houston, TX, United States of America; j Division of Medical and Scientific Relations, Alzheimer’s Association, Chicago, IL, United States of America; k Division of Public Health Sciences, Wake Forest University School of Medicine, Winston-Salem, NC, United States of America

**Keywords:** Nutrient adequacy, Supplements, Cognition, EAR, UL

## Abstract

**Background & objective:**

Consumption of diets insufficient in essential nutrients contributes to unhealthy aging. The objective of this study is to evaluate adequacy of key nutrients that reflect the food components of the Mediterranean-Dietary Approaches to Stop Hypertension Intervention for Neurodegenerative Delay (MIND) dietary pattern, one deemed important for brain health.

**Setting/design:**

Cross-sectional analyses of diets of participants in the U.S. Study to Protect Brain Health Through Lifestyle Intervention to Reduce Risk (US POINTER) trial

**Participants:**

Adults, aged 60–79 years and at risk of cognitive impairment (sedentary, suboptimal diet, other risk factors) were enrolled in the US POINTER, a phase III two-year randomized controlled trial comparing the impact of two lifestyle interventions on brain health.

**Measurements:**

Before randomization, participants completed a food frequency questionnaire (FFQ) assessing usual intake of foods and beverages, and vitamin and/or mineral supplements. Median daily nutrient intakes were expressed as a percentage of sex- and age-specific Estimated Average Requirement (EAR) or Adequate Intake (AI), and Tolerable Upper Limit (UL) criteria.

**Results:**

Of the 2111 enrolled participants, 2041 completed the FFQs (69% women). Nearly half reported multivitamin multimineral supplement use (49%, men; 49%, women). Common individual supplements included vitamins D (36%), E (25%), C (23%), and B_12_ (19%) as well as calcium (16%). Based on food alone, the proportion of participants with inadequate intakes was high across several nutrients. Supplements improved nutrient adequacy for most (except for choline, fiber and potassium) but contributed to intakes for some that exceed the UL cutoffs (vitamins A (n = 28), B_9_ (n = 134), B_3_ (n = 160) and zinc (n = 196)).

**Conclusions:**

Widespread nutrient inadequacy (especially those integral to the MIND dietary pattern) is evident among non-supplement users. Supplement use improves adequacy but raises concerns about excessive intakes that exceed ULs. Assessment of diet and supplement intake is essential to address nutrient inadequacies in at-risk older adults.

**Clinical Trial Registration Number:**

: NCT03688126, submitted 9/26/2018.

## 1 Introduction

Many Americans consume diets that do not meet national recommendations. For older adults, there are several shortfall nutrients of particular concern; these include vitamins A, B_12_, D, E, and K, folate, potassium, calcium, zinc, and fiber [1,2]. Vitamin B**_12_** supplementation is recommended for adults aged ≥50 years because of reduced bioavailability [3,4]. In addition, because naturally occurring food folates are labile and bioavailability is less than that of synthetic folic acid, cereal grains are now fortified with stable folic acid [5]. These nutrients are found in foods that are integral to healthful dietary patterns associated with neuroprotection, such as the Mediterranean, DASH, and especially, the hybrid pattern designed specifically for brain health, the Mediterranean-DASH Intervention for Neurodegenerative Delay or MIND [6].

Because older adults report dietary supplement use more than any other age group [7,8], identifying those at risk of excessive intakes through ingestion of supplements and/or fortified foods merits more attention as well. The Food and Nutrition Board of the Academies of Sciences, Engineering, and Medicine established Tolerable Upper Levels or ULs for several nutrients as the highest daily intake level unlikely to pose health risks [9]. Exceeding such amounts are associated with several adverse symptoms or conditions.

Thus, assessment of nutrient intakes from foods and beverages only may underestimate true nutrient intakes. Reports of usual intakes of food, beverages and supplements of diverse groups of older adults are limited. Recent evidence also suggests that daily ingestion of a multivitamin-mineral (MVM) supplement for 3 years improves cognitive outcomes [10]. The US POINTER trial presents a unique opportunity to assess nutrient adequacy in a diverse sample of more than 2000 older adults at risk of cognitive decline. Accordingly, this study aims to determine *whether*: (1) US POINTER participants report adequate nutrient intakes at study entry, and (2) nutrient adequacy differs between supplement users and non-users [7,8,11,12].

## 2 Methods

### 2.1 Population sample

The US POINTER trial is a phase III two-year randomized controlled trial designed to test whether two lifestyle interventions encouraging physical exercise, a healthy diet, cognitive and social activities, and health monitoring can protect brain health in older adults at risk for memory loss. Exactly 2111 individuals were enrolled from communities near five sites (North Carolina, Northern California, Chicagoland, Houston and Rhode Island). Participants were aged 60–79 years, sedentary, reported a suboptimal diet (based on a brief screener described later), and at risk for future cognitive decline based on family history of memory impairment, race or ethnicity, and/or other risk factors. The trial is registered on ClinicalTrials.gov [NCT03688126]. Details about the trial can be found in the design paper [13] and another on the topline results [14].

Suboptimal diets were defined by responses to the 14-item Mediterranean-DASH intervention for Neurodegenerative Delay (MIND) diet screener. Only participants with MIND screener scores ≤9 were enrolled in the trial. The food/beverage components that comprise the MIND pattern are based on compelling neuroprotective evidence for specific nutrients (vitamin E, folate, omega-3 fatty acids, vitamin D, vitamin B_12,_ vitamin K, fiber, potassium, choline) and foods (berries, green leafy vegetables, extra virgin olive oil) [6,15]. The brief MIND screener does not include questions on supplement use, nor does the MIND “prescription” include supplement recommendations.

### 2.2 Dietary assessment

In addition to the validated MIND diet screener (used as one of the inclusion criteria), a second instrument, a validated Rush food frequency questionnaire (FFQ) was administered prior to randomization [16,17]. The 142-item FFQ assesses *usual* intake of foods, beverages, and dietary supplements over the previous six months. Most participants completed both instruments within 3–5 weeks.

The FFQ includes detailed questions on multivitamin multimineral [MVM] supplements, vitamins E, C, B_9_ (folate), beta-carotene (provitamin A), lutein, fish oil (or omega-3 fatty acids) and three mineral (selenium, iron and zinc) supplements. For these ten items, there are questions on use [yes/no], daily dose (four choices, i.e., less than 100 IU, 100–250 IU, etc.), and duration (four choices: 0–1 year, 2–4 years, etc.). Next, there are open-ended questions on brand names of MVM supplements, other individual supplements not queried, followed by questions on frequency of food and beverage items, and specific type and brand name of cold cereals, olive oils, and margarines usually consumed. Finally, there are three open-ended questions for foods or beverages consumed frequently but not specified on the FFQ.

For each food and beverage item on the FFQ, total energy and nutrient amount in one portion size are multiplied by reported frequency and summed across all food items. Dietary intakes (including supplement contributions) and total calories are computed using the total calories and nutrients per serving obtained from the Nutrition Data System for Research (NDSR, Nutrition Coordinating Center, University of Minnesota) database, 2023 [18]. Portion sizes are described as either natural portion size [e.g., one banana] or mean portion sizes reported by the older men and women in national survey data (NHANES) collected by 24 -h dietary recalls and are based on United States Department of Agricultural National Nutrient Database and other published sources.

All FFQ processing and quality control procedures were conducted at Rush University Medical Center. FFQs with implausible caloric intakes (<700 or >4200 kcal for men, <600 and >3700 kcal for women) were excluded. Daily nutrient intakes were calculated for (1) food and beverage sources for non-supplement users, (2) food and beverages only for supplement users and (3) foods, beverages and supplements combined.

### 2.3 Supplement use

Supplement use was defined primarily based on responses to the FFQ described previously with additional information from the medication inventory completed at the same visit. This inventory included both prescription and non-prescription products. Reported items on the medication inventory included vitamins, minerals, carotenoids, omega-3 fatty acids, folic acid, MVMs, and fiber. When information on dose was missing, default values from NDSR were applied, assuming daily use. Based on the medication review with research assistants, 8.5% of data were verified by visual inspection and 53.7% were from participant-provided lists.

Total nutrient intake is calculated using contributions from food and beverages, and if consumed, amounts in reported MVM and those in individual or other combination supplements (i.e., vitamin B complex). Fiber supplements were queried during the medication review only and are not an item on the FFQ; thus, fiber supplement information was limited because dose and frequency or duration information were not specifically queried except as an over-the-counter medications (i.e., laxatives).

Anyone reporting a MVM supplement and/or individual supplement(s) was considered a supplement user; this is because questions included daily use of MVM and individual supplements, usual dose and duration of use in terms of years. Conversely, if none of these supplements were reported, the participant was classified as a non-user.

### 2.4 Dietary adequacy

Dietary Reference Intakes or DRIs are scientifically based reference values for nutrients developed by the Institute of Medicine (now the National Academies of Sciences, Engineering, and Medicine) for the general healthy populations of the US and Canada. Three DRIs are relevant here: Estimated Average Requirement (EAR), Adequate Intake or AI, and Tolerable Upper Level (UL).

The EAR is a daily nutrient amount that is thought to meet the requirement of half the healthy individuals in a group [3,5]. These differ according to age group and sex. For some nutrients, no EAR has been established yet, and then the metric is AI. Examples of nutrients with AIs include vitamin K, potassium, choline, and fiber. UL was established to assess the prevalence of higher, potentially adverse intakes. For folate and niacin, the UL reflects synthetic sources only--- potentially contributions from fortified foods and supplements. UL comparisons are not possible for some nutrients because no UL exists; these include vitamin B_12_, vitamin K, potassium, omega-3 fatty acids and fiber.

### 2.5 Other Participant Attributes

That may influence dietary intake and supplement use were acquired either by direct measurement in the clinic by trained research assistants at the US POINTER sites (weight, height, blood pressure) or self-reported on questionnaires. We dichotomized age to align with the age groups specified for the DRIs. Apolipoprotein E (APOE) genotype was assessed to identify ε4 carriers [one or more allele] and non-carriers. Education was defined as either non-college graduate or college graduate or more. Smoking status was dichotomized as never or ever; the latter was so specified because few participants were current smokers [n = 58] but there were 901 former smokers. Body mass index or BMI was based on measurements acquired at randomization and was dichotomized as overweight or obese (≥25 kg/m^2^) or not (<25 kg/m^2^). The Area Deprivation Index (ADI) was used to estimate neighborhood quality and available resources, with scores ranging from 1 [no deprivation] to 100 (greatest deprivation) [19,20]. Based on the ADI median, we dichotomized participants into two groups, ≤31 and >31.

### 2.6 Statistical analyses

Prevalence of MVM and individual supplement use between our subgroups of interest (sex, age, APOEε4, education, smoking status, BMI, and ADI) is described and compared using chi-square tests. When statistical assumptions for chi-square tests were not met (n<5), Fisher’s Exact tests were used. To account for multiple comparisons, p-values were adjusted using the Benjamin-Hochberg procedure to control the false discovery rate (FDR). Statistical significance was determined using FDR-adjusted p-values, which are reported in the tables. Concordance between FFQ supplement use with those on the medication inventory were assessed with Cohen’s kappa statistics.

Nutrient intakes [from food sources only for both non-user and supplement user) stratified by sex and age category [60−70, 71−79 y] were described as medians (and interquartile range, IQR) and were compared between non-users and users (food and supplements combined) within these age and sex groups using Mann Whitney U tests. The overall proportion of supplement users vs. non-users not meeting EARs or AIs through either food or total intake for each supplement were compared using chi-square tests or Fisher’s Exact tests, where appropriate. The proportion of participants not meeting EARs or AIs was also calculated separately for men and women and according to their age group. Median dietary and total nutrient intakes were also compared to the UL metric.

The single institutional review board (IRB) at Wake Forest University School of Medicine approved the U.S. POINTER trial with concurrence by local site IRBs. All participants provided written informed consent for the trial. De-identified data from the U.S. POINTER parent trial are available through the Global Alzheimer’s Association Interactive Network (GAAIN.org).

## 3 Results

### 3.1 Analytic sample

At baseline 2111 participants were enrolled in the trial, but 2095 US POINTER participants completed an FFQ. Of those, 2041 adults had valid FFQs (based on calorie cutoffs) and were included in the analytic sample. See Table 1. Women comprised 69.2% of the analytic sample. Participant characteristics were similar between the analytic sample and the full cohort. Most did not have an APOEε4 allele, were highly educated, never smoked, and were overweight or obese.

**Table 1:** Demographics of the baseline US POINTER sample, diet analytic sample, and those reporting multivitamin mineral supplements.^a^

	*Baseline US POINTER Sample*	Current Diet Sample	Multivitamin- mineral (MVM) Users^b^
Total number	*2111*	2041	1007
Sex			
Male	*656 [31.1]*	628 [30.8]	307 [30.5]
Female	*1455 [68.9]*	1413 [69.2]	700 [69.5]
Age			
60−70 y	*1360 [64.4]*	1323 [64.8]	646 [64.1]
71−79 y	*751 [35.6]*	718 [35.2]	361 [35.9]
APOEε4 Carrier^c^			
Yes	*659 [31.3]*	637 [31.3]	327 [32.5]
No	*1445 [68.7]*	1397 [68.7]	679 [67.5]
College Graduate or More^c^			
Yes	*1459 [69.7]*	1414 [69.8]	709 [70.5]
No	*635 [30.3]*	612 [30.2]	297 [29.5]
Smoked^d^			
Ever	*959 [45.4]*	925 [45.3]	444 [44.1]
No	*1152 [54.6]*	1116 [54.7]	563 [55.9]
Body Mass Index^e^			
≥25 kg/m^2^	*1717 [81.3]*	1665 [81.6]	827 [82.1]
<25 kg/m^2^	*394 [18.7]*	376 [18.4]	180 [17.9]
Area Deprivation Index^c^ (ADI)			
≤31	*1046 [5*0.0%*]*	1016 [50.3%]	486 [48.9]
>31	*1044 [5*0.0%*]*	1004 [49.7%]	508 [51.1]
Daily Energy Intake (kcal)	*1556* ± 593 [n = 2100]^f^	1577 ± 564	1588 ± 569

a Values in each cell reflect counts and percent of users in the specific column, e.g., 69.7% of the US Pointer participants were college graduates or more, 69.8% of the current diet sample were college graduates or more, and 30.5% of all MVM users are men, while 69.5% of MVM users are women.

b No differences between MVM users and non-users with respect to sex, age groups, APOEe4 status, college-educated status, smoking status or BMI.

c There were 7 individuals with no APOE information, 15 individuals did not report on education [dichotomous, college educated or more *vs*. no college degree], and 21 were missing ADI values.

d Smoking status, dichotomous: ever [past or current smoker] *vs*. never smoked.

e Body Mass Index at randomization.

f Total sample minus 11 who did not complete FFQs, so no caloric values were available.

### 3.2 Supplement use

Of the 2041 adults in our sample, 1601 or 78.4% were supplement users (whether from multivitamin multimineral (MVM) or individual supplements (one or more) or a combination). The greatest proportion of users (26.2%) took one supplement, but 10% reported 5 or more. Nearly half the participants (49.3%) reported MVM supplement use with similar proportions of men (48.9% of men) and women (49.5%). Reporting MVM supplements on the FFQ was highly concordant with reports from the medication review (kappa = 0.72, 95% CI 0.70, 0.76). As shown in Table 1, demographics of MVM users were like those of the entire analytic sample and the entire US POINTER sample.

Exactly 70.3% of participants reported use of supplements other than MVM (Table 2**)**. With respect to other supplements, users were more likely to be women. Sex, age, and ADI proportions of those reporting use of first five supplements on the FFQ are presented in the first part of this table. The second part [rightmost half] reflects supplements [vitamin D, calcium, vitamin B_12_, niacin, choline, and vitamin K] reported as “fill-ins” to the open-ended questions on the FFQ.

**Table 2:** Demographics of users of individual dietary supplements in the US POINTER trial at baseline n = 2041.^a^

		Supplement use in response to questions on the FFQ						Supplement use in response to open-ended questions on the FFQ					
Demographic	No.	Other vitamins/ minerals^b^	Vitamin E (mg)	Vitamin C (mg)	Zinc (mg)	Folate (mcg DFE)	Fish Oil: Omega 3 (mg)	Vitamin D (IU)	Calcium (mg)	Vitamin B_12_ (mcg)	Niacin (mg)	Choline (mg)	Vitamin K (mcg)
Total	2041	1435 (70.3)	502 (24.6)	470 (23.0)	247 (12.1)	215 (10.5)	46 (2.3)	738 (36.2)	323 (15.8)	393 (19.2)	164 (8.0)	124 (6.1)	16 (0.8)
Sex													
Men	628	**369**^c^**(58.8)**	159 (25.3)	**127 (20.2)**	69 (11.0)	65 (10.3)	12 (1.9)	**178 (28.3)**	**39 (6.2)**	108 (17.2)	56 (8.9)	39 (6.2)	3 (0.5)
Women	1413	**1066**^c^**(75.4)**	343 (24.3)	**343 (24.3)**	178 (12.6)	150 (10.6)	34 (2.4)	**560 (39.6)**	**284 (20.1)**	285 (20.2)	108 (7.6)	85 (6.0)	13 (0.9)
Age													
60-70 y	1323	920 (69.5)	319 (24.1)	309 (23.4)	164 (12.4)	135 (10.2)	29 (2.2)	480 (36.3)	191 (14.4)	245 (18.5)	110 (8.3)	84 (6.3)	12 (0.9)
71-79 y	718	515 (71.7)	183 (25.5)	161 (22.4)	83 (11.6)	80 (11.1)	17 (2.4)	258 (35.9)	132 (18.4)	148 (20.6)	54 (7.5)	40 (5.6)	4 (0.6)
Area Deprivation Index													
≤31	1016	716 (70.5)	257 (25.3)	**207 (20.4)**	123 (12.1)	101 (9.9)	25 (2.5)	392 (38.6)	147 (14.5)	193 (19.0)	79 (7.8)	56 (5.5)	12 (1.2)
>31	1004	704 (70.1)	239 (23.8)	**254 (25.3)**	122 (12.2)	112 (11.1)	21 (2.1)	340 (33.9)	170 (16.9)	194 (19.3)	82 (8.2)	66 (6.6)	4 (0.4)

a Values in each cell reflect counts and percentage of users in the specific row. Specific questions on the FFQ addressed ingestion, dose, frequency and duration of other vitamins. mineral supplements, vitamin E, vitamin C, zinc, folic acid, and fish oil. Individual supplements were also reported in response to the open-ended question and those coming from specified multivitamin, multimineral supplement brands.

b Participants reporting any vitamin and /or mineral supplements other than MVM.

c Boldface**d** values reflect significant differences in supplement use/non-use across demographic groups based on Chi square tests or Fisher's tests., i.e., for vitamin C supplements, the proportions of Vitamin C (as well as Vitamin D, and calcium) supplement users were greater for women than for men (p < 0.01).

The highest proportions of individual supplements reported include vitamin D (36.2%), vitamin E (24.6%), vitamin C (23%), vitamin B_12_ (19.2%) and calcium (15.8%). As with the general category of “other vitamins/minerals”, more women than men report use of vitamin C, vitamin D and calcium. More participants with lower ADI report Vitamin C supplement use as compared with those living in communities with higher ADI. Another notable difference (not provided on table) includes a lower proportion of overweight or obese participants who report calcium supplement use (14.6%) in contrast with those whose BMI is less than 25 kg/m^2^ (21%, p = 0.014). Among zinc supplement users, there was a lower proportion among ever smokers (9.2%) than never smokers (14.1%, p = 0.026). These comparisons were based on adjusted values for multiple comparisons.

### 3.3 Assessment of nutrient adequacy

#### 3.3.1 Nutrient inadequate intakes

In Fig. 1, we present the proportions of all US POINTER participants **not** meeting nutrient recommendations for three groups---non-users (thus, food/beverages only), supplement users (food/beverages only), and supplement users (food/beverage **plus** supplement contribution. Vitamin A intakes from foods are adequate for supplement users and non-users alike (less than 4% of non-users reported less than 625 mg per day). Less than 35% of adults reported inadequate food/beverage intake with respect to vitamins K, B_12_, and B_3_ (niacin), regardless of supplement status.

**Fig. 1:**
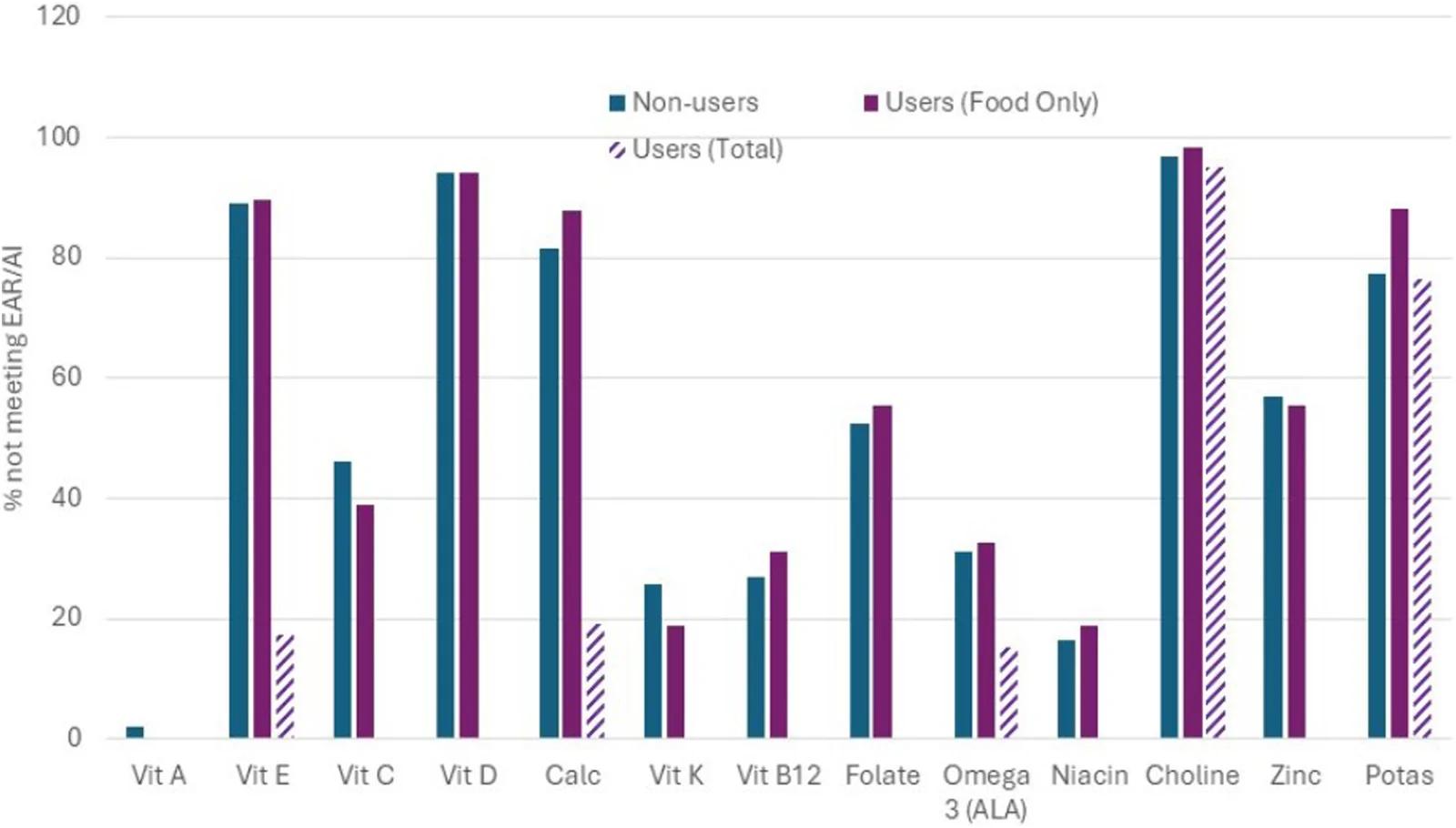
The proportion of all 2041 US POINTER participants whose nutrient intakes were **inadequate** as assessed from usual daily intakes estimated by the Rush food frequency questionnaires [FFQ]. These bars reflect three groupings, as follows: 1) non-users: adults who report usual intakes of the specific nutrient coming from food/beverages alone (no supplements reported); 2) supplement users for whom only the food contribution is being considered (excluding the supplement contribution); and 3) total nutrient intake [supplement use plus food] of the supplement user. For each nutrient, bars depict the percentage of US POINTER individuals **not** meeting the age and sex-specific Estimated Adequate Requirement [EAR] or Adequate Intake [AI]. Vit = Vitamin; Calc = calcium; Omega 3 [ALA] = alpha linolenic acid; Potas = Potassium.

Based on food and beverages alone (whether non-user or supplement user), most US POINTER participants report inadequate intakes of vitamin E, vitamin C, vitamin D, calcium, folate, omega-3 fatty acids, choline, zinc, and potassium. Supplements reduced the prevalence of inadequacy for most nutrients, but for choline, potassium.

We report usual nutrient intakes and proportions of inadequacy for women and men separately in Table 3 and Table 4, respectively. For women only (Table 3), intake from food sources usually vary minimally between non-users and users. Intakes from food only are much lower than the amounts afforded by supplements except for fish oil (n = 34), choline (n = 85) and potassium (only 13 users). As seen for all US POINTER participants, female non-users report food choices that do ***not*** meet the recommended amounts for vitamin E, vitamin D, calcium, choline, folate, potassium, zinc (60–70 years only) and fiber. Fewer women report inadequate daily fiber intakes from food (66.7% do not meet the AI) than do men (93.3%, Table 4). In contrast to females in this sample, fewer male participants report the use of individual vitamin and mineral supplements (58.8%). This contrasts with MVM use where the proportion of male and female MVM use was similar. Most male non-users consume inadequate amounts of vitamins E, C, D, B_12_, calcium, zinc, choline, folate, potassium, and fiber. In particular, 88%–100% of male participants were inadequate in terms of potassium, and 98–100% for choline. For men who use either MVM or individual supplements, intakes were adequate for most nutrients other than these nutrients.

**Table 3:** Among women, usual nutrient intakes (median, IQR) of non-supplement users (food only) and users (food only and total) and percent below the Estimated Average Requirement (EAR) based on responses to RUSH food frequency questionnaire: n = 1413.

		60−70 y (n = 933)		71−79 y (n = 480)	
Nutrient and Supplement Status	EAR	Median Intake (25, 75th Quartiles)	% below EAR^d^	Median Intake (25, 75th Quartiles)	% below EAR^d^
**Vitamin A (mcg)**	500, 500^a^	*17 supplement users*^e^		*11 supplement users*^e^	
Food only					
Nonusers (n = 1385)	√, √	2533 (1716, 3612)	1	2587 (1809, 3715)	1
Users (n = 28)	√, √	3380 (1596, 4819)	0	2238 (1774, 3779)	0
Total: Food + supplements (28 Users)	√, √	9552 (7519, 15348)	0	7728 (4638, 0277)	0

**Vitamin E (mg)**	12, 12^a^				
Food only					
Nonusers (n = 1070)	×,×	**7.1 (5.2, 9.4)**^d^	**90**^d^	**7.1 (5.1, 9.5)**^d^	**89**^d^
Users: Food only (n = 343)	×,×	7.1 (5.4, 9.2)	89	6.7 (5.4, 8.9)	92
Total: Food + supplements (343 Users)	√, √	**18.6 (12.9, 273.5)**	**19.4**	**140.9 (14.1, 275.7)**	**12.4**
**Vitamin C (mg)**	60, 60				
Food only					
Nonusers (n = 1070)	√, √	**68.1 (43.4, 103.1)**	**42**	**72.3 (44.9, 108.0)**	**41**
Users (n = 343)	√, √	76.6 (50.8, 104.4)	36	82.1 (53.5, 122.2)	30
Total: Food + supplements (343 Users)	√, √	**614.3 (555.8, 1057.5)**	**0**	**619.6 (538.2, 1058.8)**	**0**
**Vitamin D (IU)**	400, 400				
Food only					
Nonusers (n = 853)	×,×	**131 (87,189)**	95	**142 (91, 205)**	95
Users (n = 560)	×,×	142 (87, 215)	94	136 (92, 221)	94
Total: Food + supplements (558 Users)	√, √	**1152 (**1094,1241**)**	**0**	**1141 (1090, 1232)**	**0.5**
**Calcium (mg)**	1000, 1000				
Food only					
Nonusers (n = 1129)	×,×	**601 (438, 818)**	**87**	**584 (455, 806)**	**89**
Users (n = 284)	×,×	592 (448, 761)	92	626 (456, 839)	90
Total: Food + supplements (284 Users)	√, √	**1208 (1047, 1393)**	**17**	**1228 (1032, 1453)**	**23**
**Vitamin B_12_ (mcg)**	2, 2				
Food only					
Nonusers (n = 1128)	√, √	**2.7 (1.8, 3.7)**	**31**	**2.6 (1.9, 3.7)**	**29**
Users(n = 285)	√, √	2.4 (1.7, 3.4)	37	2.5 (1.6, 3.6)	35
Total: Food + supplements (285 Users)	√, √	**1002 (53.4, 1002.8)**	**0**	**1002 (63, 1003.6)**	**0**
**Vitamin K (mcg)**	90, 90^b^	*9 supplement users*^e^		*4 supplement users*^e^	
Food only					
Nonusers (n = 1400	√, √	**155 (106, 247)**	16	**142 (107, 225)**	**17**
Users(n = 13)	√, √	149 (106, 311)	22	275 (165, 383)	0
Total: Food + supplements (13 Users)	√, √	**283 (229, 411)**	0	**375 (265, 483)**	**0**
**Zinc (mcg)**	6.8, 6.8				
Food only					
Nonusers (n = 1233)	×, √	**7.0 (5.4, 8.9)**	**48**	**6.5 (5.2, 8.3)**	**55**
Users (n = 177)	√, √	6.8 (5.3, 8.7)	50	6.8 (4.8, 9.2)	49
Total: Food + supplements (177 Users)	√, √	**56 (53.2, 59.0)**	**0**	**57.0 (54.5, 61.8)**	**0**
**Choline (mg)**	425, 425^b^				
Food only					
Nonusers (n = 1328)	×,×	254 (194, 315)	96	242 (192, 306)	95
Users(n = 85)	×,×	243 (213, 303)	98	200 (172, 321)	96
Total: Food + supplements (85 Users)	×,×	266 (234, 328)	93	221 (192, 341)	92
**Niacin (mg)**	11, 11				
Food only					
Nonusers (n = 1305)	√, √	**16.1 (12.4, 20.7)**	**16**	**15.6 (11.9, 19.8)**	**20**
Users (n = 108)	√, √	16.3 (11.2, 20.7)	23	15.1 (11.4, 18.4)	24
Total: Food + supplements (108 Users)	√, √	**64.8 (59.1, 71.3**)	**0**	**64.4 (58.9, 69.8)**	**0**
**Folate/Folic Acid (mcg DFE)**^c^	320, 320				
Food only					
Nonusers (n = 1263)	×,×	**306 (240, 389)**	**55**	**314 (239, 408)**	**52**
Users (n = 150)	×,×	317 (251, 414)	52	285 (222, 348)	67
Total: Food + supplements (150 Users)	√, √	**1089 (937,1222)**	**0**	**1012 (772, 1148)**	**0**
**Potassium (mg)**	2600, 2600	*6 supplement users*^e^		*7 supplement users*^e^	
Food only					
Nonusers (n = 1400)	×,×	2143 (1694, 2683)	71	2137 (1693, 2687)	73
Users (n = 13)	×,×	1856 (1353, 2327)	83	1820 (1236, 2504)	86
Total: Food + supplements (13 Users)	×,×	1955 (1452, 2426)	83	1919 (1335, 2603)	71
**Dietary Fiber- Food Only (g)**^f^	21, 21	*0 supplement use on FFQ*^e^		*0 supplement use on FFQ*^e^	
	×,×	17.5 (13.5, 22.9)	66	17.5 (13.4, 22.4)	67
**Fish Oil: Omega 3 (g)**	1.1, 1.1	*23 supplement users*^e^		*11 supplement users*^e^	
Food only					
Nonusers (n = 1379)	√, √	1.5 (1.1, 2.1)	24	1.5 (1.1, 2.1)	25
Users (n = 34)	√, √	1.3 (0.9,1.7)	39	1.6 (1.4, 2.1)	9
Total: Food + supplements (34 Users)	√, √	1.6 (1.1, 2.1)	22	2.2 (1.6, 2.3)	0

a The first cutoff number is for 60 to 70-year-old adults, the second for those 71 and older. In this column depicting the EAR, we present “√” s when the proportion of participants not meeting the EAR is less than 50% (stated the other way, more than 50% of adults meet the recommendation), and “ꭓ”s, where more than 50% do not meet the recommendations (EAR/AI). In the next columns we provide median intakes and 25th and 75th percentiles of each nutrient followed by the actual percentage of adults whose reported intakes were below EAR or AI.

b This is AI or Adequate Intake, not an EAR.

c DFE = dietary folate equivalents.

d Boldfaced values differ significantly non-users vs. total (food + supplement) users by Chi-square or Fisher’s tests. Based on food contribution alone, supplement users and non-users do not differ with respect to the % not meeting the EAR or AI unless noted.

e Where the number of supplement users are noted for each age group, no statistical comparisons were deemed appropriate.

f No queries on fiber supplements are present on the FFQ, and hence, estimates of frequency and dose were not possible.

**Table 4:** Among men, usual intake (median, IQR) of non-supplement users (food only) and users (food only and total) and percent below the Estimated Average Requirement (EAR) based on responses to RUSH food frequency questionnaire: n = 628.

		60−70 y (n = 390)		71−79 y (n = 238)	
Nutrient and Supplement Status	EAR	Median Intake (25, 75th Quartiles)	% below EAR^d^	Median Intake (25, 75th Quartiles)	% below EAR^d^
**Vitamin A (mcg**)	625, 625^a^	*4 supplement users*^e^		*7 supplement users*^e^	
Food only					
Nonusers (n = 617)	√, √	**2023 (1366, 3017)**	**4**	**2153 (1404, 3247)**	**4**
Users (n = 11)	√, √	2626 (2173, 3204)	0	2625 (1555,7128)	0
Total: Food + supplements (11 Users)	√, √	**8933 (7216,** 13986**)**	**0**	**9025 (**5025,18378**)**	**0**
**Vitamin E (mg)**	12, 12				
Food only					
Nonusers (n = 469)	×,×	**7.3 (5.5, 9.8)**^d^	**87**^d^	**7.7 (5.5, 9.7)**^d^	**90^d^**
Users: Food only (n = 159)	×,×	7.6 (5.8, 10)	91	6.8 (4.9, 9.3)	87
Total: Food + supplements (159 Users)	√, √	**17.4 (13.2, 272.1)**	**15**	**20.7 (12.1, 272.9)**	**23**
**Vitamin C (mg)**	75, 75				
Food only					
Nonusers (n = 501)	×,×	**67 (48.2, 103.5)**	**56**	**66.7 (40.8, 107.3)**	**56**
Users (n = 127)	×,√	64.2 (45.3, 109.6)	57	81.1 (46.7, 120.1)	46
Total: Food + supplements (127 Users)	√, √	**681.9 (544.2, 1059.6)**	**0**	**696.1** (**566.7, 1067.6)**	**0**
**Vitamin D (IU)**	400, 400				
Food only					
Nonusers (n = 450)	×,×	**160 (101, 221)**	**93**	**161 (111, 245)**	**94**
Users (n = 178)	×,×	153 (90, 221)	93	160 (104, 226)	97
Total: Food + supplements (178 Users)	√, √	**1165 (1104, 1258)**	**0**	**1182 (1105, 1248)**	**0**
**Calcium (mg)**	800, 1000				
Food only					
Nonusers (n = 589)	×,×	**690 (484, 914)**	**65**	**733 (557, 931)**	**79**
Users (n = 39)	×,×	696 (509, 968)	59	732 (474, 906)	75
Total: Food + supplements (39 Users)	√, √	**1296 (1027, 1530)**	**11**	**1269 (1011, 1394)**	**25**
**Vitamin B_12_ (mcg)**	2, 2				
Food only					
Nonusers (n = 520)	×,×	**3.0 (2.3, 4.2)**	**20**	**3.2 (2.2, 4.4)**	**20**
Users (n = 108)	×,×	2.9 (2, 3.7)	24	2.8 (2.4, 4.3)	9
Total: Food + supplements (520 Users)	√, √	**1001.7 (52.9, 1003.3)**	**0**	**1002.3 (55.2,1003.5)**	**0**
**Vitamin K (mcg)**	120,120^b^	*3 supplement users*^e^		*0 supplement users*^e^	
Food only					
Nonusers (n = 625)	√, √	124 (87, 173)	47	129 (87, 179)	45
Users (n = 3)	√, n/a	163 (98, 259)	33		
Total: Food + supplements (3 Users)	√, n/a	263 (198, 359)	0		
**Zinc (mcg)**	9.4, 9.4				
Food only					
Nonusers (n = 559)	×,×	**7.7 (6.2, 10.1)**	**69**	**7.7 (6, 9.6)**	**74**
Users (n = 69)	×,×	7.0 (5.5, 9.4)	70	8.1 (7, 10.4)	69
Total: Food + supplements (69 Users)	√, √	**55.5 (27.5, 59.9)**	**0**	**58.9 (54.9, 74)**	**0**
**Choline (mg)**	550, 550^b^				
Food only					
Nonusers (n = 589)	×,×	269 (225, 352)	98	283 (220, 345)	99
Users (n = 39)	×,×	281 (229, 332)	100	285 (226, 326)	100
Total: Food + supplements (39 Users)	×,×	302 (250, 353)	100	305 (247, 347)	100
**Niacin (mg**)	12, 12				
Food only					
Nonusers (n = 572)	√, √	**18.8 (14.5, 23.5)**	**13**	**18.5 (14.2, 23)**	15
Users (n = 56)	√, √	18.3 (13.9, 23.6)	9	16.8 (12.6, 19.9)	14
Total: Food + supplements (56 Users)	√, √	**68.3 (62.2, 78.4)**	**0**	**68.1 (62, 76.2)**	0
**Folate/Folic Acid (mcg DFE)**^c^					
Food only	320, 320				
Nonusers (n = 563)	×,×	**316 (249, 424)**	**52**	**337 (255, 420)**	**44**
Users (n = 65)	×,×	311 (216, 457)	55	317 (270, 418)	50
Total: Food + supplements (65 Users)	√, √	**1114 (999, 1284)**	**0**	**1099 (**911,1241**)**	**0**
**Potassium (mg)**	3400, 3400^b^	*3 supplement users*^e^		*1 supplement user*^e^	
Food only					
Nonusers (n = 624)	×,×	2215 (1849, 2935)	88	2345 (1889, 2863)	90
Users (n = 4)	×,×	2535 (1529, 2727)	100	3316	100
Total: Food + supplements (4 Users)	×, √	2634 (1628, 2826)	100	3415	0
**Fiber (g)**^f^	30, 30	*0 supplement use on FFQ*^e^		*0 supplement use on FFQ*^e^	
Food only					
Nonusers (n = 628)	×,×	17.7 (14.2, 22.5)	94	17.7 (12.7, 22.4)	92
**Fish Oil: Omega3 (g**)	1.6, 1.6	*6 supplement users*^e^		*6 supplement users*^e^	
Food only					
Nonusers (n = 616)	√, √	**1.7 (1.2, 2.3)**	43	1.6 (1.1, 2.2)	51
Users (n = 12)	√,×	2.7 (2.1, 2.8)	17	1.5 (1.3, 1.8)	67
Total: Food + supplements (12 Users)	√, √	**2.9 (2.8, 3.5)**	17	2.0 (1.7, 2.5)	17

^c^DFE = dietary folate equivalents.

a The first cutoff number is for 60 to 70-year-old adults, the second for those 71 and older.

b This is AI or Adequate Intake, not an EAR.

d Boldfaced values for non-users and the total values of supplement users differ significantly by Chi-square or Fisher tests. Based on food contribution alone, supplement users and non-users do not differ with respect to the % not meeting the EAR or AI. unless noted.

e Where number of supplement users are noted per each age strata, no statistical comparisons were deemed appropriate.

f No fiber supplements were reported on the FFQ and hence, estimates of frequency and dose were not possible.

#### 3.3.2 Excessive nutrient intakes

In Fig. 2 we illustrate the proportion of supplement users for whom excessively large supplement doses pose potential adverse risk. More than 95% of users report amounts of vitamin A and niacin (vitamin B_3_) that exceed the UL and warrant attention (vitamins A (n = 38), B_3_ (n = 160)). Folate (vitamin B_9_)-containing supplements were reported by 215 adults and 134 reported doses that exceeded the UL. Zinc supplements were reported by 247 adults, 196 of whom reported consuming amounts far greater than the UL.

**Fig. 2:**
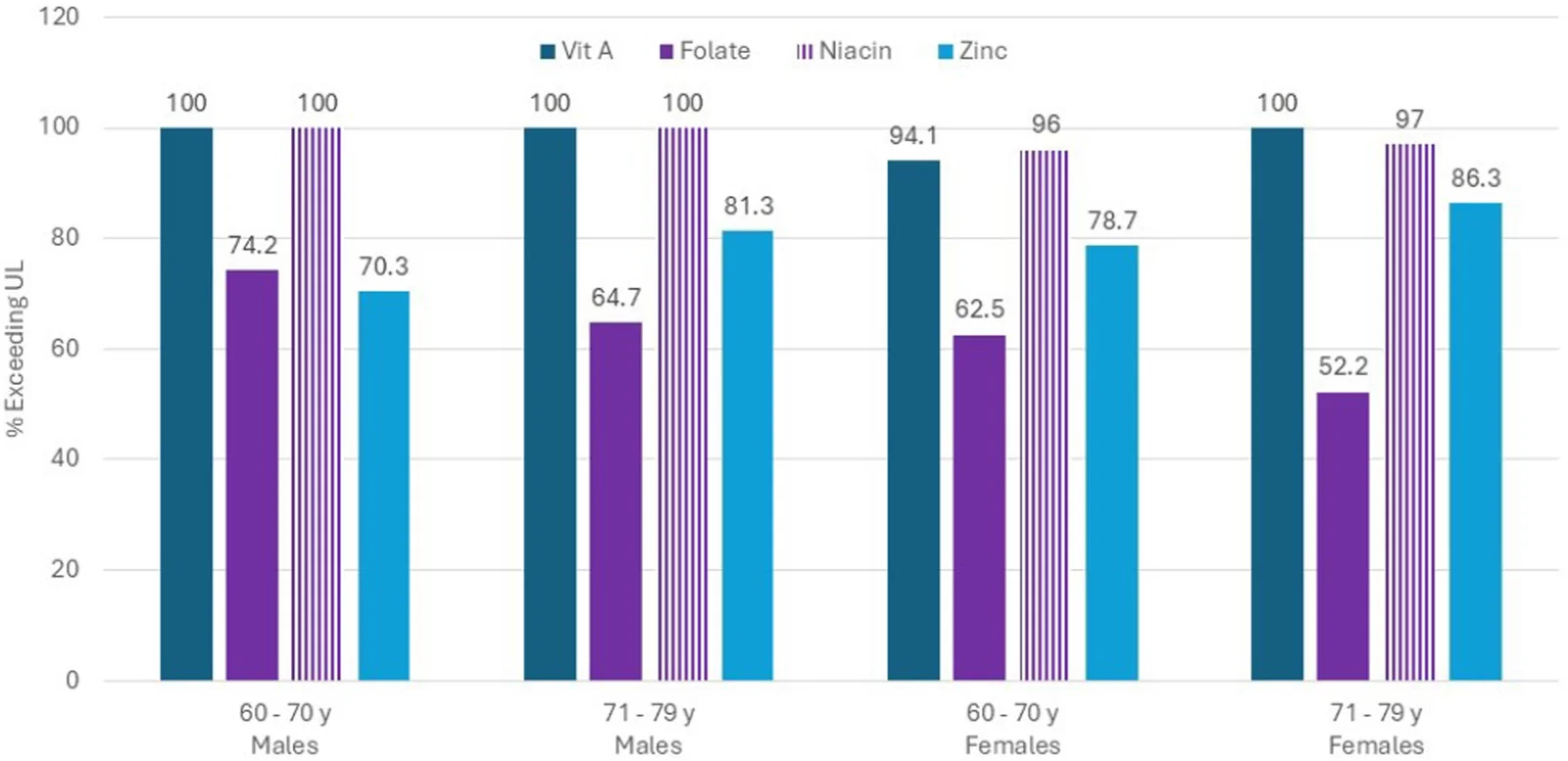
The proportion of US POINTER supplement users whose intakes **exceed** the tolerable upper limit [UL] for safe and adequate intake. Bars reflect clusters for men and women by age group. Responses are estimated by the Rush food frequency questionnaires [including fill-ins] and expressed as nutrient intakes in relation to the UL appropriate for age and sex of the participant. Only those nutrients with ULs are presented. Tolerable ULs are as follows: Vitamin A, 3000 mcg; Folate, 1000 mcg; Niacin, 35 mg; and Zinc, 40 mg.

## 4 Discussion

Nutrition experts encourage adequate nutrition from food [2]. Nutrient-dense food choices are important when energy needs decrease with age [21]. Our findings suggest that food choices alone are often insufficient to meet nutrient recommendations for older adults in US POINTER. Supplement intake appears to improve nutrient adequacy for many individuals. Inadequate potassium intake, which is also an indicator of vegetable intake [22,23] was observed among non-users and supplement users alike. Many MVM supplements do not contain potassium, but those that do typically provide only 80 mg potassium [24] --- far less than the recommended amount; the AI for potassium is 3400 mg per day for men; 2600 mg for women [6]. Another essential nutrient that was inadequate for many U.S. POINTER participants was choline. Choline is available in dietary supplements containing choline only, in combination with B-complex vitamins, and in some MVM products [24]. Amounts of choline in dietary supplements typically range from 10 mg to 250 mg. Yet supplements appear to contribute minimally to choline recommendations in the present study sample. In a recent NHANES report, choline intakes were also found to be inadequate for men and women (average, 396 mg for men, 260 mg for women) [25]. Based on another study based on 24 -h recall data from NHANES 2003–2018, 70% of adults, ages 71 years and older, did not meet the EAR for calcium based on food/beverages alone. Once supplements were included, however, the proportion of inadequacy was reduced to 39.7% [26]. The proportion of individuals with excessive intakes [in relation to calcium UL [2000 mg/day]] was not provided. Among US POINTER participants, calcium inadequacy exceeded 80% with diet alone but when supplements were included, proportions of inadequacy were much lower ---11% to 25%.

Dietary information for participants in cohort studies typically is acquired through use of FFQs. However, few reports describe participant adequacy of nutrient intakes or quantify the contribution of supplements to such. The latter is particularly surprising since the older adults exhibit the highest prevalence of supplement use [7,8,27]. In the Health and Retirement Study (HRS), a nationally representative cohort of 6045 middle-aged and older participants, 84.6% were regular supplement users, with MVM being the most common (57.5%) [28]. As in the present study, vitamin D, calcium, fish oil, vitamin C and Vitamin B_12_ supplements were common. However, nutrient adequacy was not reported for the HRS participants. In the Multiethnic Study of Atherosclerosis (MESA), median intakes of calcium, potassium and vitamin C derived from FFQs (including supplement use) were reported for more than 6000 adults ages 45–84 years and compared to EAR and UL cutoffs [29]. When supplements were included, many adults had intakes that exceeded the UL for calcium [15%, MESA; 2.5%, US POINTER], and for vitamin C [6.6%, MESA; 0%, US POINTER]. Similar to US POINTER participants, potassium intakes were inadequate (less than 10% met the AI), and supplement use contributed minimally. Nutrient intakes of Women’s Health Initiative Long Life Study participants (n = 5732 women, mean age 79 years) were also compared to the DRIs in a recent report [30], but supplement use was not described.

The DRI committee recognizes that supplements may fill nutrient gaps by enhancing the likelihood of meeting recommended intake [9]. National data on nutrient intakes exceeding ULs are limited, out of date or not reported for many micronutrients [26,[29], [30], [31]]. Adverse effects of chronic vitamin A ingestion (greater than 8000 micrograms retinol activity equivalents per day (RAEs)) may include dry, cracked skin, hair loss, brittle nails, fatigue, loss of appetite, liver abnormalities (elevated liver enzymes, fibrosis) and possibly reductions in bone mineral density with increased risk of hip fracture [32]. The UL is considerably lower, 3000 RAEs, than 8000 RAEs, though many ingest as much more (Table 3, Table 4). Too much folic acid may be linked to increased prostate cancer risk; high doses derived from fortified foods and supplements can also cause nausea and irritability [24]. The most common side effect of consuming too much niacin is flushing and can occur at doses of as low as 30 mg per day. Other symptoms are itching, nausea, headache, vomiting, light-headedness, and epigastric pain [33]. The tolerable UL for zinc has been set at 40 mg/day primarily to counter nausea or abdominal pain with consumption of higher amounts, but also because of the deleterious impact on copper nutriture [34]. Large dose supplement use warrants examination in the context of other concomitant medications or supplements because of potential interactions. It is critical that health providers inquire not only if dietary supplements are consumed but also dose and frequency.

Strengths to this study include a large, diverse sample and the use of a validated FFQ with detailed supplement assessment. Unlike many other FFQs, Rush FFQ has open-ended queries for MVM supplement brand names. Corroboration of MVM supplement use was confirmed by the medication interview. Open-ended questions also address other supplements taken daily that are not present on the FFQ. There are also open-ended queries for brand names of cereals, margarines, and oils. There is the opportunity to list additional food or beverages consumed frequently but not present on the FFQ. Such features foster greater specificity of respondent nutrient intakes, thereby enhancing accuracy of usual nutrient intakes by older adults as compared to many other FFQs.

Our findings should be interpreted with some limitations in mind. First, to be eligible for US POINTER, participants had to exhibit a suboptimal diet quality based on a brief MIND diet screener. Because the 14-item screener does not include questions on supplement use, total nutrient intakes could not be estimated nor the likelihood of inadequacy or excessive intakes. Second, it is difficult to know how comparable the USPOINTER sample is to other cohorts of older adults where some nutrition information has been published, since these cohorts were originally designed to track cardiovascular morbidities, and because of differences in cognitive measures reported. Third, reporting usual intake of foods/beverages on the FFQ is subject to possible recall bias [like all dietary methods] and because certain foods or beverages may not specifically queried on the FFQ. Fourth, data provided are without adjustment for potential confounders, such as age, sex, education and kilocalories.

These results underscore the need for careful evaluation of total nutrient intake among older adults. Nutrient estimates should include not only usual food and beverage choices but also supplements in terms of dose and frequency.

## 5 Conclusion

Few groups describe risk of inadequacy at both ends of the nutrition spectrum—too little (less than the EAR or AI) or too much (greater than the UL) among older adults. Supplement use may introduce risks alongside benefits.

Widespread nutrient inadequacy is evident among non-supplement users in the US POINTER sample. Fewer supplement users exhibit nutrient inadequacy, though the possibility of adverse impact on individuals consuming nutrients exceeding UL merit further attention. More research on impact of intakes exceeding the UL is indicated in light of these findings.

Comprehensive assessment of dietary and supplement intake is critical to address possible nutrient inadequacies among older adults, especially among at-risk groups like that of US POINTER. At a minimum, we suggest that health care providers use a validated dietary screener and additional questions on usual supplement use [including dose and duration].

## CRediT authorship contribution statement

**Christy Tangney:** conceptualization, data curation, investigation, methodology, project administration, writing -original draft, review and editing. **Marjorie Howard:** formal analysis, methodology, validation, visualization, writing- review and editing. **Jennifer Ventrelle:** data curation, investigation, writing- review and editing. **Puja Agarwal:** conceptualization, methodology, writing- review and editing. **Katelyn R Garcia:** formal analysis, methodology, writing-review and editing. **Sarah M Graef:** methodology, writing-review and editing. **Thomas Holland:** data curation, writing-review and editing. **Masun Jackson:** writing-review and editing. **Jeffrey A Katula:** conceptualization, writing-review and editing. **Joseph C Masdeu:** data curation, writing-review and editing. **Talitha Baszlie:** data curation, writing-review and editing. **Kristie Miller:** data curation, writing-review and editing. **Rose A Trevino-Whitaker:** data curation, writing-review and editing. **Heather M Snyder:** conceptualization, funding acquisition, investigation, methodology, writing-review and editing. **Laura D Baker:** data curation, funding acquisition, investigation, methodology, project administration, supervision, writing-review and editing. **Mark A Espeland:** methodology, supervision, writing-review and editing.

## Declaration of Generative AI and AI-assisted technologies in the writing process

AI-assisted technologies (ChatGPT) were used to format references only.

## Funding

This work was supported by the Alzheimer’s Association [19-611541] to Drs. Laura D. Baker, Mark A. Espeland, Heather Snyder, and Rachel Whittmer.

## Declaration

During the preparation of this work the author used CHATGPT to format references. After using this tool, the author reviewed and edited the content as needed and takes full responsibility for the content of the published article.

## Data availability

Anonymized data supporting the findings of this study are available from the corresponding author.

## Declaration of competing interest

None.
